# Blood Metagenome in Health and Psoriasis

**DOI:** 10.3389/fmed.2020.00333

**Published:** 2020-09-16

**Authors:** Nikolay Korotky, Mikhail Peslyak

**Affiliations:** ^1^Department of Dermatovenereology, Pirogov Russian National Research Medical University, Moscow, Russia; ^2^Antipsoriatic Association “The Natural Alternative”, Moscow, Russia

**Keywords:** bacterial DNA, bacterial products, intestinal permeability, metagenome, microbiome, non-host DNA, phagocytes, psoriasis

## Abstract

A survey and analytical assessment of the results of fundamental works on studying blood metagenome (set of all non-human DNA) is carried out. All works on determining bacterial DNA concentration in the whole blood of healthy people are reviewed. Detailed comparison of characteristics of 16S rRNA test (hereinafter 16S-test) and whole metagenome sequencing test (hereinafter WMS-test) is carried out and published in Supplement S1. One of main goals of this review is to identify the drawbacks and mistakes which the studied works contain, particularly to emphasize the crucial importance of determining total concentration of bacterial DNA for comparing patients' metagenomes with those of healthy people as well as for comparing patients' metagenomes with each other. Controlling the level and composition of contamination is equally important. The absence of high-quality contamination control at each step (or at certain steps) of the research significantly reduces the reliability of achieved results. The given review is the first attempt to analyze and systematize the results of blood metagenome studies, whose number has increased considerably in the last few years. The review has been carried out as part of preparation for implementing a project on complex studying metagenomes of whole blood and skin biopsies of psoriatic patients.

## Introduction

Epidermis self-renewal is a regular process. New cells are born in the basal layer and they mature, vary, migrate outside and form an external horny layer. They then die away and exfoliate. The standard duration of an epidermis cell life (renewal period) for areas of skin with an average thickness is 20–25 days. Psoriasis accelerates self-renewal an cells live 4–10 days (1–3). Cells migrating outside have no time to differentiate and are not as functional. Psoriatic plaques have a red shade, they are tender, covered by white flakes due to intensive loss of cells, and are much thicker.

Psoriasis is not contagious and there is various types of psoriasis: vulgaris or plaque (L40.0), flexural or inverse (L40.83-4), erythrodermic (L40.85), pustular (L40.1-3, L40.82), guttate (L40.4). Codes of diseases are given according to ICD-10. Chronic plaque psoriasis is the most frequent type (more than 80% of total number of cases). Up to 15% of psoriatics also suffer from psoriatic arthritis (L40.5).

Psoriasis strikes about 2% of the population (incidence varies by country). The disease appears after birth or in extreme old age. Psoriasis is a chronic disease, so there are periods of aggravation and remission. Sometimes there is no cause for period change and occasionally aggravation can be decreased as a result of treatment. Serious psoriasis can result in disability. A psoriasis course is similar in men and women. Afro-Americans, Indians, Chinese and Japanese people suffer from psoriasis less frequently and Eskimos do not suffer from psoriasis at all (4–6).

Psoriasis is registered in the “Online Mendelian Inheritance in Man” at number OMIM^*^177900. Psoriasis is a disease with hereditary predisposition: concordance of uniovular twins is 70%. If one parent suffers from psoriasis, children are diagnosed with the disease in 15–25% of cases; if both parents suffer from psoriasis, children are diagnosed with the disease in more than 40–60% of cases. The interrelation of allele HLA-Cw^*^0602 (chromosome 6p21) and psoriasis of the first type which is characterized by early beginning is proved. This allele is found in more than 60% of psoriatic patients (hereinafter PP) and not more than 15% of healthy persons (hereinafter HP). Locuses of other chromosomes have weaker interrelations. Psoriasis cannot begin only in the presence of genetical deflections. External exposure is necessary for beginning and maintenance of psoriasis. Infections, skin traumas, stresses, reaction to medications, climatic changes, and other causes can provoke onset of psoriasis or its aggravation.

Psoriasis is frequently accompanied by general diseases, including metabolic syndrome, diabetes mellitus of the II type, coronary heart disease, arterial hypertension, pathology of hepatobiliary system, depression, and some others (7, 8).

## Metagenomic Sequencing

The blood metagenome has been frequently identified for its various fractions, both for patients with various diseases, and for healthy people. It has been primarily identified in plasma or serum (the majority of works), but also in buffy coat, platelets and erythrocytes, whole blood or in neutrophils (9–12) (Table 1).

**Table 1:** Blood metagenome researches.

**Patients**	**Biomaterial**	**Test**	**Determining concentration**	**Contamination control**	**nhDNA (particularly bacDNA)**	**Study (country), notes, reference to bioproject**
**PP (psoriatic patients) and HP**						
19 PP	Plasma	16S	No	Yes	bacDNA in 9 out of 19 PP	[(13), USA], PP with psoriatic arthritis. Metagenome was not identified.
15 PP, 12 HP	Monocytes	16S, 18S	Yes	Yes	bacDNA in all	[(14), Japan]. Relative level in PP is 1.5–2 times higher than in HP. Metagenome was not identified. bacDNA concentration was estimated in reference to concentration of gene human glyceraldehyde-3-phosphate dehydrogenase (GAPDH).
20 PP, 12 HP	Plasma	16S	No	Yes	bacDNA in all PP, in no HP	[(15), UK]. Metagenome was not identified.
54 PP, 27 HP	Serum	16S	No	No	bacDNA in 16 out of 54 PP, in no HP	[(16), Spain] – article, nuanced in (17).
52 PP	Serum	16S	No	No	bacDNA in 13 out of 52 PP	[(17), Spain]. Based on the same material as (16)
**Non-psoriatic patients and/or HP**						
50 HP	Leukocytic mass	16S	Yes	No	bacDNA in all	[(18), Canada]. Metagenome was not identified.
3280 persons	Leukocytes	16S	Yes	No	bacDNA in all	[(19), France] - quantitative 16S. Qualitative 16S was used for 42 people only. Patient group common with (20), including those with diabetes.
3936 persons	Leukocytes	16S	Yes	No	bacDNA in all	[(20), France], concentration assessment only for Eubacteria and Proteobacteria phylum in general. Metagenome was not identified. Patient group common with (19), including those with cardiovascular diseases.
80 patients and 40 HP	Plasma	16S and WMS	Yes	Yes, NTC	bacDNA in all	[(21), India], WMS-test was performed for 3 patients and 3 HP.QIAamp DNA blood mini kit was applied.
50 patients, 50 HP	Plasma	16S RT-PCR	No	No	bacDNA in 14 out of 50 patients, in 2 out of 50 HP	[(22), Japan], patients with type 2 diabetes. Tests of blood plasma were carried out in addition to tests of feces. Limited set of primers was used. Metagenome was not identified.
78 patients and 10 HP	Plasma	WMS	No	No	nhDNA in all	[(23), China], post-surgery patients, all nhDNA was studied, culture was performed.
30 HP	Whole blood and 3 fractions	16S	Yes	Yes, NTC, 0.05%	bacDNA in all	[(9), France], HP – donors (postprandial blood tests). Increased bacDNA concentration provided by (24) method. NucleoSpin Blood L was applied.
2 groups of patients (Spain - 37, Italy - 71)	BC fraction and whole blood	16S	Yes	No	bacDNA in all	[(25), France], each of the two patient groups was divided into two parts (without fibrosis, with fibrosis). BC – Buffy coat.
30 HP, 90 patients	Plasma	WMS	Yes – for all DNA only	Yes (not published)	nhDNA in all	[(26), Germany], all nhDNA was studied, patients with sepsis and post-surgery patients. Culture was also performed. ENA: PRJEB13247
9 patients	Non-host cells in whole blood	WMS	No	Yes	nhDNA in all	[(27), Sweden], acute leukemia and suspected sepsis. Before sequencing, consecutive enrichment was performed (Molysis, NebNext). All nhDNA was studied. Concentration was determined only for cell-free DNA.
62 patients and 23 HP	Whole blood	16S	No	Yes	bacDNA in all	[(28), Poland], patients with sepsis, DNA isolation procedure is based on their own research (29).
188 patients, 1,351 samples	Plasma	WMS	No	Yes, NTC	nhDNA in all	[(30), USA], patients after transplantation (heart, lungs, bone marrow), and pregnant women. Numerous blood sampling from the same patients under observation. NTC was formed of sterile culture of human cells.
28 HP	Whole blood	16S	No	Yes, dH_2_O	bacDNA and fungal DNA in all	[(31), Bulgaria], culturing, and comparing of whole blood metagenomes for different blood groups were carried out.
56 patients, 20 HP	Whole blood	16S	Yes		bacDNA in all	[(11), USA, France], patients with alcoholic syndrome, DNA isolation procedure is similar to (9).
50 patients and 12 HP	Whole blood, neutrophils	16S	Yes	Yes	bacDNA in all	[(10), China], patients with pancreatitis. For the first time metagenome of blood neutrophils is identified.
45 HP and 45 patients + 57 HP and 58 patients	Leukocytic mass	16S	Yes	Yes, NTC = 6, 0.14%	bacDNA in all	[(12), China], patients with Parkinson's disease. Results on concentration raise doubts.
5 HP and 5 patients	Plasma	16S	No	Yes	bacDNA in all	[(32), United Kingdom], patients with asthma, culturing, RNA-seq.
20 patients and 20 HP	Leukocytic mass	16S	Yes	No	bacDNA in all	[(33), USA, France], patients with chronic renal failure, but without diabetes. Technique of DNA isolation is similar to (9).
50 patients and 100 HP	Plasma	16S	No	Yes	bacDNA in all	[(34), China], patients with type 2 diabetes.
18 patients and 10 HP	Whole blood	16S	No	No	bacDNA in all	[(35), USA], patients with celiac disease. Fecal metagenome is also obtained, and its comparison with blood metagenome is made.
324 patients and 402 HP	Serum	16S	No	No	bacDNA in all	[(36), Korea], patients with cirrhosis or with liver carcinoma.
88 patients and 13 HP	Serum	16S	No	No	It is not clear	[(37), China], patients with gastric carcinoma.
**NCS1 research project**						
30 PP, 10 HP	Whole blood	WMS	Yes	Yes	nhDNA in all	[(38), Russia], blood sampling 3 h after food intake, preliminary elimination of hDNA, all nhDNA. Similar tests (of phagocytes) of psoriatic biopsies. Their complex study.

Bacterial DNA (hereinafter bacDNA) concentration in plasma is more than three orders lower than in buffy coat: 1.5 × 10^4^ (16S copies)/ml against 4.2 × 10^7^ (16S copies)/ml (9). It is due to this that in many cases bacDNA in plasma was not found at all or, even if it was found, it was only in some patients. The fact is that all bacterial products (as well as any non-host ones, including non-human DNA – hereinafter nhDNA), which get into blood, are constantly utilized.

For the purposes of discussion, their utilization can be subdivided into two main ways: phagocyte-dependent (binding, endocytosis) and phagocyte-independent. Phagocyte-independent utilization is assured by degrading enzymes, proteins and antibodies, which connect bioproducts in complexes subsequently brought out of blood flow through elimination organs (primarily kidneys and liver). Utilization of non-host bioproducts can be considered completely phagocyte-independent if their degradation occurs without phagocyte participation. Utilization of non-host bioproducts is phagocyte-dependent if they are endocyted (binded) by blood phagocytes and at the time of endocytosis (binding) can still be recognized as non-host. Inside phagocytes utilization the process of non-host bioproducts definitely continues, though for some time all of them can still be recognized (Supplement S4).

This very process occurs at destruction of blood phagocytes and subsequent identification of all DNA – a certain part of it proves to be nhDNA. nhDNA concentration in blood phagocytes and it appears to be considerably higher than in plasma. I.e., at each timepoint most of nhDNA present in blood is in blood phagocytes (9).

Studies of the presence of bacDNA in PP and HP blood began as far back as in the last century. Let us pass over to a detailed review of the results.

In (13) blood plasma of patients with psoriatic arthritis was investigated; in 9 PP out of 19, bacDNA of *Streptococcus pyogenes, Str. agalactiae*, and *Str. pneumoniae* were found. Their presence was determined by the PCR method with specific primers for these species. Metagenome was not identified.

In (14) on several primers for 16S rRNA the presence of bacDNA in blood monocytes was identified in 15 PP and 12 HP (bacDNA was found in all PP and HP). It was discovered that in PP it is considerably higher than in HP. Concentration of bacDNA amounted to ~3.1 (16S copies)/monocyte for HP and ~ 5.8 (16S copies)/monocyte for PP on average [(14), Figure 2].

Similar comparison was made on several primers for 18S rRNA (average excess for PP compared to HP is 1.5 times). The authors assumed that the main source of bacDNA was intestine microbiome. Metagenome was not identified.

In (15) by 16S-test the presence of bacDNA in plasma of peripheral blood of 20 PP and 12 HP was detected; it was found in all PP and in no HP. In 17 PP the discovered bacDNA was identified as belonging to bacteria from genera *Streptococcus* or *Staphylococcus* [(15), Table 1]. Their presence was determined by the PCR method with specific primers for these genera. Metagenome was not identified.

In a short article (16) it is reported that by 16S-test bacDNA in blood serum is found only in 16 PP out of 54 and in none of 27 HP. Another study of the same material has been published recently, which also contains results of 16S-test of fecal metagenome of 52 PP (17).

The fact that bacDNA was not found in blood plasma in HP, and frequently in most PP (whereas researches of whole blood were not conducted), made it difficult to make any assertions about the role of its presence in blood in psoriasis pathogenesis. But in recent years there has been considerable improvement of research techniques: human DNA (hereinafter hDNA) elimination methods, enhancing of accuracy and reliability of results in detecting small quantities and, most importantly, phenomenal depreciation of whole metagenomic sequencing (WMS-tests). For detailed comparison of 16S-tests and WMS-tests (see Supplement S1).

Let us outline the main results of these works.

In (18) concentration of bacDNA and hDNA in blood and saliva of donors was studied, their percentage in all DNA isolated from whole blood and saliva, respectively, was determined. Metagenome was not identified.

In (19, 20) the results of long-term research involving more than 5,000 people were summarized. The main objective of this research was identifying the reasons and conditions provoking diabetes. The research was initiated by D.E.S.I.R. Study Group. Among numerous examinations conducted within 9 years (at 3-year intervals) there was quantitative 16S-test of blood leukocytes. The procedure of receiving bacDNA with the maximum concentration in sample was elaborated. In (19) we can find comparison of results for patients with diabetes and without one, in (20) – for patients with cardiovascular diseases and without them. In (19) for a small part of patients with diabetes (*n* = 14) and control group without one (*n* = 28) qualitative 16S-tests were carried out, which made it possible to determine bacterial relative presence (hereinafter representation) to within genus. In (20) metagenome was not identified.

In (21) 80 patients with cardiovascular diseases and 40 HP were examined. Blood plasma was used as biomaterial. For all patients 16S-test was used, and for 3 patients and 3 HP WMS-test was additionally applied.

In (22) 50 patients with type 2 diabetes and 50 HP were examined. By 16S RT-PCR test fecal microbiome and blood plasma microbiome was studied with a limited set of primers (for 21 species, genus or phylum of bacteria). 16S RT-PCR test of blood plasma was qualitative (yes/no), the presence of bacDNA in blood plasma was registered in 14 patients and in 2 HP. Metagenome was not identified. The search of correlations between bacterial genera found in feces and in blood was not carried out.

In (23) 78 post-surgery patients and 10 HP were examined. WMS-test for blood plasma was carried out, accompanied by bacterial culture [(23), Table 1]. The presence of nhDNA was determined (bacteria to within species, fungi and viruses), mapping was carried out on reference catalogs of NCBI Genome. The majority of reads was mapped on human genome (95.6% on average), <1% of the rest was mapped on genomes of particular bacteria, fungi or viruses.

In (9) 16S-test of whole blood as well as its fractions was carried out on 30 HP (donors) in order to detect bacDNA. The method elaborated and tested by the authors earlier was applied (24). It turned out that bacDNA is found in all HP and also that its greater part is found in buffy coat, i.e., in the fraction of leukocytes and platelets (93.7%), and the smaller part is connected with the fraction of erythrocytes (6.2%) and blood plasma (0.03%). This is mainly bacDNA of Gram(–) bacteria of phylums *Proteobacteria* (87%) and *Bacteroidetes* (classes *Sphingobacteriia, Bacteroidia* and *Flavobacteriia*) (2.5%), but also mainly Gram+ bacteria of phylums *Actinobacteria* (6.7%), *Firmicutes* (class *Bacilli*) (3%).

This research did not aim at establishing the sources of bacDNA origin in blood. Therefore, it remained unclear:

- If living or degraded bacteria which appeared in blood were source of the discovered bacDNA;
- How bacDNA was connected with leukocytes, platelets and erythrocytes;
- Where bacteria and/or bacterial products containing bacDNA got into blood from.

Note that bacDNA concentration found in blood plasma−1.4 × 10^4^ (16S copies)/ml on average – is of the same order with bacDNA concentration found in the control test of one of the reagents−1.5 × 10^4^ (16S copies)/ml.

The results for whole blood have sufficient reliability as bacDNA concentration amounted from 1.8 × 10^7^ to 7.6 × 10^7^(16S copies)/ml). On average 4.2 × 10^7^ (16S copies)/ml. Direct correlation between bacDNA concentration and leukocyte concentration in blood was demonstrated.

Expressed in terms of leukocytes bacDNA concentration amounted to ~5.7 (16S copies)/leukocyte [(9), Supplementary Figure 2a]. And as phagocytes are responsible for utilizing non-host bioproducts in blood, it corresponds to ~8.5 (16S copies)/phagocyte. It exceeds the concentration in ~3.1 (16S copies)/monocyte for HP (14), if only because in this study postprandial blood was investigated.

The results of this research within genus are only given for the main pathogens: *Acinetobacter* ~6 × 10^5^ (16S copies)/ml; *Corynebacterium* ~4 × 10^6^ (16S copies)/ml, *Escherichia* or *Shigella* ~1.5 × 10^5^ (16S copies)/ml (16S-test does not distinguish between these two genera), *Pseudomonas* ~ 1.5 × 10^6^ (16S copies)/ml, *Staphylococcus* ~1.7 × 10^5^ (16S copies)/ml, *Stenotrophomonas* ~2.3 × 10^5^ (16S copies)/ml. 16S average concentration found in buffy coat is specified (at the rate on 1 ml of whole blood). Genus of the bacteria *Shewanella* ~10^4^ (16S copies)/ml is found only in plasma [(9), Figure S3].

The authors assume that the main source of bacDNA income into blood is intestine microbiome and the bacDNA distribution for phylums similar to the one in mucous biopsies of the small intestine taken from Treitz ligament in HP is valid (39). Higher total bacDNA concentration in blood (compared to other studies) is connected with the fact that HP were donors and took food and drink before blood donation (as is often recommended to donors, and also according to the conditions of this research).

It is known that food intake leads to rapid growth of small intestine microbiome (more than by 50 times with celiac disease and with irritable bowel syndrome). This, in its turn, causes temporary growth (presumably the same) of bacterial products income in systemic blood flow (40). First it happens in connection with microbiome growth (waste products), and then (in the process of himus move on the gastrointestinal tract) because of microbiome reduction (dying off products). In dying off products peptidoglycan (hereinafter PG), lipopolysaccharide (hereinafter LPS) and other pathogen-associated molecular patterns (hereinafter PAMP) constitute a considerable proportion.

The method designed in (24) was applied to 16S-tests of buffy coat (patients in Spain) and whole blood (patients in Italy) (25). Each group of patients was divided into two parts (with or without fibrosis). Venipuncture was carried out after 12-h abstinence from food. A considerably smaller amount in comparison with (9) bacDNA concentration – about 2.4 × 10^3^ (16S copies)/ml for groups without fibrosis and 1.5–2 times more for groups with fibrosis — was found.

In (26) the following three groups were examined: patients with sepsis (*n* = 60), patients after abdominal surgery (*n* = 30) and HP (*n* = 30). DNA which is found free in blood plasma (cell-free DNA) was studied. Its concentration was determined by means of Qubit dsDNA HS Assay Kit (Life Technologies). Then the WMS-test was carried out, and reads belonging to hDNA were analytically excluded (96–98% on average).

The other reads were mapped to within species with the use of NCBI RefSeq (reference genomic DB) [(26), Table 1]. As a result, averagely from 2.3 to 4.2% of not excluded reads were mapped, for 12 HP — averagely 3.5% of not excluded reads, which corresponds to averagely 0.064% of all reads. Information of nhDNA representation was eventually obtained.

In (27) only patients with suspected sepsis (*n* = 9) were examined. nhDNA of non-host cells found in blood was studied. Before sequencing, consecutive enrichment of biomaterial was performed. First, MolYsis Complete 5 kit was applied. With the help of this kit the following is carried out in succession:

- All host blood cells in the sample collapse (when this occurs, the majority of non-host cells are not affected);
- Non-host cells are isolated from the sample (whereas practically all contents of host cells are removed);
- Non-host cells collapse, and from them nhDNA is isolated.

Next, for the samples received through such preparation, additional enrichment of nhDNA by decreasing hDNA concentration (NebNext microbiome enrichment) was performed. Then, the WMS-test was carried out and reads belonging to hDNA (79%) were analytically excluded; other reads were mapped to within species with the use of NCBI Genome. They succeeded in mapping only 0.07% of reads. nhDNA of bacteria, viruses and fungi in correlation with the patients' condition was discovered.

In (28) 62 patients with sepsis and 23 HP were examined, by 16S-test whole blood was studied, and information about bacDNA representation within genus was obtained. The technique of DNA isolation from whole blood was based on their own research (29). Extra tests with samples of no template control (hereinafter NTC) were carried out, which demonstrated composition of contamination (pollution of samples and/or reagents).

In (30) a large group of transplantation patients (heart, lungs, bone marrow) as well as 32 pregnant women (188 patients in total) were examined. Blood sampling was carried out repeatedly at different stages of transplantation and pregnancy (1,351 samples in total). WMS-test was applied to study plasma (extracellular, circulating) DNA. There are no published data on its concentration. The emphasis in the research is placed on detecting and studying not mapped nhDNA (i.e., such DNA which cannot be compared to any known genomes of bacteria, archaea, viruses, etc.).

Ninety-fifth percentage of reads underwent quality control, and out of these 99.55% on average were mapped on reference human genome (GRCh38), i.e., only 0.45% on average remained for mapping on non-host reference. Only averagely 1% of not excluded were mapped on reference containing genomes of nearly 8,000 species of known bacteria, archaea, viruses, fungi and other eukaryotes (i.e., 0.0045% on average of all reads). About 1,800 species (out of nearly 800 genera) were found in all blood samples.

Chart SF15 (30) shows quantitative characteristic of the mapped at the level of domains and phylums: *Bacteria* (528 in total), including such phylums as *Actinobacteria* (248), *Firmicutes* (183), *Proteobacteria* (86) and *Deinococcus-Thermus* (2); *Eukaryota* (145 in total), including such phylums as *Ascomycota* (96), *Chordata* (7), *Bacillariophyta* (5) and *Streptophyta* (4) and *Viruses* (100 in total). More detailed information (for example, on genera or species) is absent from the paper and appendices.

The results in (30) are distinctly different from (26), primarily in a very high proportion of reads mapped on human genome (99.55% against 96–98%) and also in a lower proportion of reads mapped on non-host reference (0.0045% against 0.064% of all reads on average).

It might be connected with the process of blood plasma isolation. According to (30) it occurred by more intensive centrifugation (1,600 g, 10 min. + 16,000 g, 10 min.) while in (26) it was only (292 g, 10 min. + 1,000 g, 5 min.), which led to the removal of most large nhDNA fragments from plasma.

In this study manifold contamination control was applied. More specifically, some samples of NTC were formed of hDNA received from definitely sterile cultures of human cells. A similar approach to forming sample of NTC will be implemented in this project (38).

In (31) healthy persons are examined (28 people, with all the blood groups equally represented). Whole blood was cultivated with a special technique for low concentrations. All the samples demonstrated cultural growth. 16S-tests were carried out both for cultivated and initial samples of whole blood. For the first time fungal metagenome was identified by ITS2. DNA isolation was performed by the researcher's own method with the subsequent use of two standard kits. bacDNA concentration was not determined; distilled water was used as control samples. Taxons found in control samples were eliminated from metagenome of the main samples.

In initial samples of whole blood the maximum representation was demonstrated by *Rhizobiales* and *Sphingomonadales* orders (over 90% in total), representation of *Bacillales* order amounted to appr. 2.3%. The results at the level of families and genera are not given in the study, though the accuracy of the 16S-test enables us to do so. The results on whole blood metagenome do not correlate with the results in (10), they are not so exact and not analyzed thoroughly enough.

In (11) patients with alcoholic syndrome of different severity (*n* = 56) and HP control group (*n* = 20) were examined. The method of DNA isolation from whole blood was the same as in (9). bacDNA concentration was subsequently determined and 16S-test was applied. bacDNA concentration for HP amounted to 66 (16S copies)/(DNA ng) on average [(11), Figure 1A]. NucleoSpin Blood kit was applied for DNA isolation from blood (average DNA yield amounted to 25,000 ng/ml). It is therefore possible to estimate bacDNA concentration on 1 ml of whole blood in 1.65 × 10^6^ (16S copies)/ml = 66 (16S copies)/(DNA ng) × 25,000 ng/ml. It is <4.2 × 10^7^ (16S copies)/ml on average for postprandial whole blood in (9), probably because blood sampling was carried out on an empty stomach. From the information of HP whole blood metagenome it follows that representation of *Streptococcaceae* family amounted to ~2.8% [(11), Supplementary Figure 3A].

In (10) patients with pancreatitis (*n* = 50) and HP control group (*n* = 12) were examined. Metagenome was studied by the 16S-test, whole blood as well as previously isolated blood neutrophils were used as biomaterial. bacDNA concentration was determined only in whole blood and for 12 HP averaged 1.38 × 10^8^ (16S copies)/ml. Blood sampling was carried out on an empty stomach and blood was immediately (before DNA isolation) processed by Qiagen. Mechanical homogenization, similar to (9) and (11), was not applied. Right after processing by RLT buffer, QIAamp DNA Mini Kit (Qiagen) was applied.

Blood metagenomes (both of whole blood and of isolated neutrophils) of patients and control group are analyzed in detail at the level of phylums, classes, families and genera. It is demonstrated that 80–90% of blood metagenome are constituted by the bacteria present in fecal metagenome. The authors suggest that intestine microbiome is the main source of bacterial product income into systemic blood flow.

It may be assumed that the highest bacDNA concentration in HP whole blood (compared to other studies) is achieved due to the immediate use (right after blood sampling) of RLT buffer. Its use proved to be more effective (compared to mechanical homogenization) not only for disrupting blood cells, but also for delaying bacDNA degradation processes.

In (12) two groups of patients with Parkinson's disease were examined. For the first group (*n* = 45), their spouses (*n* = 45) were selected as HP control group. One of the criteria to be selected for the first group was continuous residence in the given region for at least 20 years. Such restrictions were not applied for the second group of patients with Parkinson's disease (*n* = 58) and for HP control group (*n* = 57). DNA isolation was made from leukocytic mass [similar to (19) and (20)]. bacDNA concentration in leukocytic mass was determined only for the first group of patients and HP. As well as in (11), it was determined in the form of 16S copy quantity found in 1 ng of all DNA. bacDNA concentration for HP averaged 7.78 × 10^3^ 16S copies on 1 ng of all DNA [(12), correct Table 2], which corresponds to averagely 1.17 × 10^8^ (16S copies)/(ml of whole blood).

Whittle et al. (32) studies the blood of five patients with pre-existing asthma and of 5 HP. Plasma was used as biomaterial for culturing and RNA/DNA isolation. The time of blood sampling is not indicated. The culturing tested positive for eight samples (four patients and 4 HP). Reducing contamination level during venipuncture was ensured by eliminating the first test tube. Ultraclear water (molecular biology grade water) was used as NTC. The authors consider intestine, oral and skin microbiomes to be the main sources of live bacteria and bacterial product income into blood.

Shah et al. (33) studies blood of patients with chronic renal failure and HP. The blood was taken from 2010 to 2017 and stored in a cryobank. As a result of detailed study of patient records, blood samples of 20 patients and 20 HP were selected. The (9, 11) technique was applied; leukocytic mass was used as biomaterial for DNA isolation. The time of blood sampling is not indicated; concentration was identified as the number of 16S copies found in 1 ng of all DNA. For 20 HP bacDNA concentration averaged 122 (16S copies)/(DNA ng), which corresponds to appr. 3.0 × 10^6^ (16S copies)/(ml of all blood). This is almost twice as high as in (11) and 10 times lower than in (9) for 30 HP. That is probably due to the fact that sampling of blood, stored in the cryobank, was performed on an empty stomach. Contamination level was not evaluated in this study.

Qui et al. (34) studies blood of 50 patients with type 2 diabetes and 100 HP. Plasma was used as biomaterial for DNA isolation. Blood sampling was carried out on an empty stomach; bacDNA concentration was not determined. The main part of blood metagenome was made by two classes: *Alphaproteobacteria* (average representation for HP − 56.9% and for patients −56%) and *Betaproteobacteria* (40.8 and 41.7% respectively), including two genera, *Sphingomonas* (51.6 and 51.1%) and *Variovorax* (36.9 and 37.3%) [(34), Tables S2 and S5].

This is essentially different from the results in (9) (respective classes constitute 54.9 and 21.9%, respective genera – below 1%) and (10) (respective classes − 9 and 7%, respective genera — below 1%). *Sphingomonas* and *Variovorax* genera with representation as in (34) were not previously found in blood and skin metagenomes of HP, and if they were, that was only in some samples and in a relatively small percentage [(41), skin metagenome, under 10% of sites, representation lower than 3%].

Qui et al. (34) does not say anything about measures of reducing contamination by skin commensals during venipuncture. It can be assumed that blood from the first test tube was used for DNA isolation, whereas these two genera are the most widespread skin commensals of palmar forearm of the local population.

In (35) metagenomes of whole blood and fecal metagenomes were identified for 18 patients with celiac disease and 10 HP. Comparison of metagenomes was carried out. Concentration was not determined; measures to reduce and control contamination are not indicated. In HP blood metagenome, the following phylums demonstrated maximum representation: *Proteobacteria* (42.3%), *Firmicutes* (32.1%), *Actinobacteria* (8.4%), and *Bacteroidetes* (5.9%) [(35), Figure 1A]. The authors register a considerable decrease of genus *Bifidobacterium* representation at patients (under 10 OTU on average) compared to HP (300 OTU on average), which correlates with the information on the positive role of this genus' bacteria in intestine microbiome as well as on its possible role in preventing and reducing celiac disease severity. The authors regard intestine microbiome as the main source of bacterial product income into blood, and hence they believe that blood metagenome can to a large extent characterize intestine microbiome composition.

In (36) blood metagenome was identified for 324 patients with cirrhosis or carcinoma of liver, and for 402 HP. Serum was used as biomaterial, and notably it underwent specific preprocessing before DNA isolation (including 40-min boiling at 100°C). bacDNA concentration was not determined; measures to reduce and control contamination are not indicated. In HP blood metagenome the following phylums demonstrated maximum representation: *Firmicutes* (~40%), *Proteobacteria* (~26%), *Actinobacteria* (~12%), *Bacteroidetes* (~7%), *Verrucomicrobia* (~1.5%), and including genus *Pseudomonas* (10.1%), *Streptococcus* (5.8%), *Bacteroides* (~4.2%), *Bifidobacterium* (4%), *Acinetobacter* (2.2%), *Staphylococcus* (2%), *Klebsiella* (2%), *Enterococcus* (2.2%), *Faecalibacterium* (~2.2%), *Akkermansia* (1.9%), and *Prevotella* (1.2%) [(36), Figure 3 and Table S1]. The authors point out that representation of genera *Bifidobacterium* and *Streptococcus* at HP was considerably higher than at patients. HP selection was carried out only on the basis of assessing their state of health at the time of blood sampling, whereas no questions were posed about taking antibiotics or any other drugs potentially influencing blood metagenome. Comparing blood metagenomes of patients and of HP has made it possible to reveal a number of essential differences. They are regarded as the result of patients' intestine microbiome changes.

In (37) blood metagenome was identified for 101 samples (88 patients with gastric carcinoma and 13 HP). Only these sample results out of 311 samples were kept for analysis after sequencing (261 patients and 50 HP). Low quality of sequencing results is mentioned as the main reason for this. Serum of blood taken on an empty stomach was used as biomaterial. bacDNA concentration was not determined; measures to reduce and control contamination are not indicated. In HP blood metagenome the following phylums demonstrated maximum representation: *Proteobacteria* (60.8%), *Actinobacteria* (25.8%) and *Firmicutes* (10%), including genus *Pseudomonas* (10.8%), *Sphingomonas* (~10%), *Propionibacterium* (~9%), *Corynebacterium* (~7%), *Hydrogenophilus* (~5%), *Geobacillus* (~5%), *Acinetobacter* (~3%) [(37), Figures 2c,d].

A recent extensive review in (42) “The Healthy Human Blood Microbiome: Fact or Fiction?” is a retrospective review of studying the presence of bacterial products and live bacteria in HP blood. The authors analyze early works where culturing was applied as well as recent works where 16S-tests were also carried out (including many of the listed above).

The main conclusion made by the authors is that the presence of bacterial products (including bacDNA) in the blood of HP is normal. The authors suppose that there is always a certain amount of live bacteria in HP blood. They consider intestine microbiome to be the main source of their income (as well as that of bacterial products).

Table 1 contains a brief description of the researches listed above. Note should be taken that bacDNA concentration in whole blood (leukocytic mass) in HP was determined only by the 16S-test and only in four of the studies mentioned above (Table 2). There is a wide scatter of results. This can be partly explained by the following variations: by preparation for blood donation (obligatory meals for donors, 12-h fasting for most groups of patients); by fractions (whole blood, leukocytes, buffy coat); by methods of DNA isolation (43); by algorithms of read processing; by methods of measurement (assessment) of bacDNA concentration. Safety of nhDNA (including bacDNA) contained in blood phagocytes at the time of blood sampling is definitely affected by the storage and transportation time and conditions, as well as pretreatment before DNA isolation. Blood phagocytes (until they are destroyed) continue degradation of earlier endocyted non-host products (including nhDNA). The rate of this degradation depends on many factors (transport environment, temperature, etc.).

**Table 2:** bacDNA concentration in HP whole blood.

**Study**	**Okubo et al. (14)**	**James et al. (18)**	**Païssé et al. (9)**	**Puri et al. (11)**	**Li et al. (10)**	**Qian et al. (12)**	**Shah et al. (33)**
Country	Japan	Canada	France	USA, France	China	China	USA, France
Biomaterial	Monocytes	Leukocytic mass	Whole blood (and other fractions)	Whole blood	Whole blood	Leukocytic mass	Leukocytic mass
HP (healthy persons) and patients	12 HP and 15 PP	50 HP (donors)	30 HP (donors)	20 HP (and others)	12 HP (and others)	45 HP (and others)	20 HP (and others)
Time of blood sampling	ND	ND	After drink and meal	ND	After fasting	ND	ND
Preprocessing of samples before DNA isolation			Homogenization	Homogenization	RLT buffer	Phenol/chloro-form	Homogenization
Whole blood (16S copies)/(ml of whole blood)		4.29 × 10^7^ (recalculation)	4.20 × 10^7^	1.65 × 10^6^ (recalculation)	1.38 × 10^8^	1.17 × 10^8^ (recalculation)	3.05 × 10^6^ (recalculation)
Determination of bacDNA concentration (qPCR)	-	V3, special primers, 194–amplicon	V3-V4, EUBF, EUBR, 467–amplicon	V3-V4, universal primers, ND	V3, 357f/518r, 162–amplicon	V3-V4, EUBF, EUBR, 467–amplicon	qPCR (V3-V4, EUBF, EUBR, 467–amplicon)
Average bacDNA *E. coli* weight proportion from all DNA in whole blood (recalculation)	0.022%	0.060%	0.059%	0.002%	0.194%	0.164%	0.004%
(16S copies)/monocyte (14) (16S copies)/leukocyte (9) (recalculation)	HP − 3.1; PP − 5.8		5.7				
Concentration range	HP: 1.5–7.5	0–0.48% (in % of weight)	1.8 × 10^7^- 7.6 × 10^7^	±50%	8 × 10^7^- 2 × 10^8^	?	Q1 = 2.9 × 10^6^, Q3 = 3.7 × 10^6^
Contamination control			NTC		NTC ≥ 5	NTC = 6	
Contamination level			0.048% (average)		0.003% (maximum)	0.138% (average)	

Contamination can also affect sequencing results. Contamination can occur during venipuncture, from reagents or from the medium in which tests are carried out. It can come from the performer, it can be accidental, etc. Thus, the results in (34) confirm the necessity of eliminating the first test tube (and ideally the second one as well!) during venipuncture.

Using whole blood as biomaterial instead of plasma is also preferable since bacDNA concentration in whole blood exceeds its concentration in plasma by more than three orders (9). The fact remains that identical accidental (or inevitable) contamination for whole blood samples results in an error which is three orders lower than for plasma samples.

For more detail about contamination and ways of its control and record (44–46).

In the table, for each work there is a note on whether contamination control was exercised (if there is no relevant information in the paper, it is presupposed that there was no such control) (Table 1).

The review of works is executed in chronological order that allows to see if blood metagenome research methods for the last 20 years were improved. In total such works it has appeared 27 (Table 2). From them, only 18 works in which metagenome completely was defined. Supplement S3 contains table in which these 18 works (and two more) are grouped in two key parameters: biomaterial sampling type (serum or plasma or whole blood, phagocytes) and sequencing technique (16S-test or WMS-test). In the same place comparison of their results is executed.

## bacDNA Concentration in Healthy Whole Blood

We have collected all published statistically significant results of researches in which bacDNA concentration in whole blood of healthy people was determined (Tables 1, 2).

For the project it is important to achieve the greatest possible bacDNA concentration and, consequently, the highest bacDNA representation in all DNA isolated from whole blood. As a result, after enrichment performance by NebNext Microbiome Enrichment (NME) bacDNA representation will become even higher and, consequently, the percentage of nhDNA reads in output (after WMS-test) will increase.

As it follows from the series of works by French researchers (9, 19, 20, 25), the maximum bacDNA concentration in whole blood is achieved in the postprandial term. However, neither breakfast menu nor blood sampling time after breakfast in (9) are specified, bacDNA concentration in whole blood before and after food intake for the same patients was not compared.

There are several studies devoted to measuring LPS (and other substances) concentration in blood after food intake (47–52). Dynamics of postprandial bacDNA concentration in whole blood is similar to dynamics of postprandial LPS concentration (measured in plasma, though) (47). In the performance of project's tasks statement and results of these researches will be taken into account.

bacDNA concentration in whole blood is affected by preprocessing, namely by how soon after blood sampling the degradation processes of bacterial products in and out of blood cells will be terminated. For this very reason in (9, 11) triple mechanical homogenization was applied. However (judging by the results), the use of RLT buffer (10) is desirable, which made it possible to achieve higher bacDNA concentration in fasting blood compared to postprandial blood (9): 1.38 × 10^8^ against 4.2 × 10^7^ (16S copies)/(ml of whole blood).

## Conclusion

Let us enumerate the main drawbacks present in some of the researches listed above.

Without determining total bacDNA concentration, comparing metagenomes of patients and healthy people becomes virtually pointless. Comparing several examinees' metagenomes makes sense only if their total bacDNA concentrations differ very little.

If determining total bacDNA concentration has been done, in the absence of contamination control there is still a problem of reliability of the received results, since it still remains unknown for which bacDNA concentration is considerably higher than contamination level (these values are significant), and for which bacDNA concentration does not exceed contamination level (these values are insignificant).

Païssé et al. (9) demonstrated for the first time that bacDNA concentration in whole blood (leukocytic mass) is three orders higher compared to its concentration in plasma. However, in most studies of blood metagenome carried out after the publication of this work, only plasma (or serum) metagenome was identified. Thus, values of bacDNA concentration were considerably closer to contamination level in absolute values.

In the same study, bacDNA concentration in postprandial blood was determined for the first time. There is every reason to suppose that it is significantly higher than in fasting blood (at least one order higher). Studying metagenome of postprandial whole blood makes it possible to improve the reliability of information on minor components of blood metagenome, primarily due to reducing the influence of contamination.

Reducing contamination level and reliable evaluation of its level necessitate elaboration of research protocol, including sampling, storage and transportation of biomaterials as well as including a sufficient number samples of NTC in the protocol (at all its steps).

In several studies contamination composition was determined, but its level was not estimated. Such an approach is irrational as merely knowing contamination composition does not enable us to consider its influence on metagenome of the main samples. It is a mistake to eliminate any bacDNA from metagenome of the main samples just because they were found in samples of NTC. Information on bacDNA concentration both in the main samples and in samples of NTC is indispensable for correcting metagenome of the main samples.

This review is made as part of preparation of NCS1 project (38) in which the main hypotheses of systemic YN-model of psoriasis pathogenesis will be tested (see Supplement S5).

Up to the present, blood metagenome of PP has been studied only by 16S-test (plasma and serum) (15, 17) whereas metagenome of PP whole blood has never been identified. In (14) only monocytes were studied, notably without metagenome identification. The method of whole genomic sequencing (WMS-test) has never been applied to studying metagenome of whole blood (Supplement S3).

On the basis of the results of implementing NCS1 project, for the first time answers to the following questions will be received:

- Does severity of psoriasis correlate with concentration of any nhDNA in whole blood?.
- Does severity of psoriasis correlate with any PAMP concentration in blood? (53).
- Does severity of psoriasis correlate with increased macromolecular small intestine permeability?

To achieve this, for the first time (for PP and HP) (Supplement S3):

- Parameters of fragment distribution of bacDNA, found in DNA-samples from whole blood are determined.
- Whole blood metagenome is identified by whole metagenomic sequencing method.
- nhDNA concentration in whole blood is determined (for the first time for PP).
- Macromolecular small intestine permeability is determined by bacDNA-test.

In the process of preparing and implementing the project, measures will be taken to minimize contamination level. Using samples of NTC will enable us to obtain complete and reliable information about the level and composition of contamination. This information will be considered for correcting metagenome of the main samples. Using WMS-tests will make it possible to determine metagenome composition to within species (and, if need be, to within strains). This will enable us to answer the questions raised within the project with sufficient reliability.

## Author Contributions

MP and NK: conceptualization and methodology. MP: formal analysis, visualization and writing. All authors contributed to the article and approved the submitted version.

## Conflict of Interest

The authors declare that the research was conducted in the absence of any commercial or financial relationships that could be construed as a potential conflict of interest.

## Figures and Tables

**Table:** 

	**Description**
**16S**	16S ribosomal RNA
bacDNA	Bacterial DNA
hDNA	Host DNA (here – human DNA)
HP	Healthy person
**LPS**	Lipopolysaccharide
Metagenome	Set of all nhDNA (non-host DNA, i.e., in this project of non-human), contained in biomaterial.
nhDNA	Any non-host DNA (here non-human) – from bacteria, archaea, fungi, plants, helminths, viruses, phages, etc.
**NTC**	No Template Control. Control samples which do not contain DNA what is supposed to be found in the main samples. Are applied to determination of contamination level and concrete contaminants.
**OTU**	Operational taxonomic units
**PAMP**	Pathogen-associated molecular patterns (in particular LPS, PG, bacDNA) (53)
**PG**	Peptidoglycan
PP	Psoriatic patient
Representation	Relative presence (percentage share) something in biomaterial (sample).
WMS	Whole metagenome sequencing (shotgun)

***Bold** indicates common abbreviations*.

## Supplementary Material

Supplement S1 Comparative characteristics of 16S and WMS-tests.

Supplement S2 Resources of metagenomic research and sequencing.

Supplement S3 Main researches classification and comparison.

Supplement S4 Income of bacterial products into blood flow from small intestine.

Supplement S5 YN-model of psoriasis pathogenesis. Partial description.
