# Supplementary material for: Blood Metagenome in Health and Psoriasis
Source: Front Med (Lausanne). 2020 Sep 16;7:333. doi: 10.3389/fmed.2020.00333 (PMC7524894; doi:10.3389/fmed.2020.00333)
Supplement: Supplement S1 — Comparative characteristics of 16S and WMS-tests. [file Data_Sheet_1.PDF]

Blood metagenome in health and psoriasis.

Supplements.

Nikolay Korotky [1], **Mikhail Peslyak** [1,2]

[1] Department of Dermatovenereology, Pirogov Russian National Research Medical University, Moscow, Russia
[2] Antipsoriatic Association "The Natural Alternative", Moscow, Russia

*** Correspondence:** Mikhail Peslyak mikp2000@gmail.com

Content

S1. Comparative characteristics of 16S and WMS-tests 2

S2. Resources of metagenomic research and sequencing 5

S3. Main researches classification and comparison. 6

S4. Income of bacterial products into blood flow from small intestine. 8

S5. YN-model of psoriasis pathogenesis. Partial description. 9

References for supplements 10

S1. Comparative characteristics of 16S and WMS-tests

	Characteristics	16S	WMS	Sources
	**Basic**			
1	Opportunity to detect any species, including uncultivated ones.	Yes	Yes	
2	Identifying DNA of both live and dead (partly degraded, including degraded DNA) organisms. It is impossible to identify separately.	Yes	Yes	
3	Detecting non-bacterial DNA (from eukaryotes, archaea, viruses, phages, fungi, plants, parasites, etc.).	No	Yes	Jovel 2016, Meisel 2016, Ranjan 2016
4	Level of high-quality taxon classification. 16S-test cannot technically ensure identification to within species for ~ 42% of genera, as trans-species coincidence of amplikon sequence is higher than 97%. Only WMS-test gives an opportunity to identify bacDNA to within species (strain) (Jovel 2016).	Phyla +++<br>Genus ++<br>Species -+<br>Strain -	Phyla +++<br>Genus +++<br>Species +++<br>Strain +-	Frey et al., 2015, Meisel et al., 2016, Ranjan et al., 2016, Tyakht et al., 2014
5	Representation (phylums, genera, species) is determined.<br>To determine concentrations by representations, an additional test of initial biomaterial is carried out. qPCR (Glassing et al., 2016, Païssé et al., 2016) and dPCR (Bhat et al., 2016, Tan et al., 2015) are most often applied to determine total bacDNA by one of the universal 16S rRNA sites (Nakatsuji et al., 2013). "The internal standard method" – adding a specific amount (about 1% of the totally expected amount) of DNA of a characteristic bacterium to biomaterial – is also applied. It is such a bacterium whose bacDNA definitely cannot be present in this biomaterial (Tan et al., 2015). Before sequencing, concentration of all DNA is always determined. This can be enough to determine concentration by WMS-test results.	Yes	Yes	Bhat et al., 2016, Glassing et al., 2016, Grumaz et al., 2016, Païssé et al., 2016, Tan et al., 2015
6	Genome coverage. For WMS-test, possible even for DNA with low representation (depends on coverage depth).	One or several sites in 16S rRNA	Uniform coverage of all genome.	Ranjan et al., 2016
7	Identifying specific genes.<br>Due to this, WMS-test results can be interpreted with precision up to within strains on marker unique genes (Jovel et al., 2016).	No	Yes	Ferretti et al., 2017, Ranjan et al., 2016
8	Identifying genes of resistance to antibiotics (resistome).	No	Yes	Frey et al., 2015

	Characteristics	16S	WMS	Sources
9	Identifying genes of virulence (pathogenicity).	No	Yes	Meisel et al., 2016
10	Functional classification of the detected species, discovery of new genes.	No	Yes	Jovel et al., 2016
11	Information for the choice of medicines.	+	++	Frey et al., 2015
	**Additional characteristics and disadvantages**			
12	Quantity of genera (species) of bacteria for which 16S rRNA sequence (fully or partly, for 16S-test) or strain genome sequence (for WMS-test) is established. Data for Feb 14, 2019.	~ 3360000 (RDP, r11); ~ 6800000 (SILVA, r132)	~ 186000 (including ~13500 whole) Genome	
13	Quantity of bacteria species (for 16S-test with inaccurate classification) found in one biomaterial (excrements) by increasing the size of libraries (up to 3.2×10^7).	2050	4100	Ranjan et al., 2016
14	Microbiome variety found in one biomaterial on three various metrics.	Lower	Higher	Ranjan et al., 2016
15	Test-sensitivity is limited by possible contamination. Test samples of NTC are necessary to assess contamination level. It is also essential to take constant measures to reduce this level.	Yes	Yes	Glassing et al., 2016
	**WMS-test vs 16S-test.**			
16	When the pathogen is unknown, when more than mere identification is required (identifying the strain, assessing pathogenic load and resistance to antibiotics). In case of mixed infections (e.g. at mixed sepsis), 16S-test often results in mistakes and has weak repeatability.	No	Yes	Frey et al., 2015
17	Opportunity to detect DNA of any species, and not only that which is included into a pre-determined list. The best 16S tests of sepsis diagnostics SepsiTest (over 345 species of bacteria and fungi) and IRIDICA (over 1000 pathogens, out of production since 2017) are unable to identify pathogen presence, if it is not from their list.	No	Yes	Frey et al., 2015, Stevenson et al., 2016
18	Identifying genomes of new, previously undetected species.	No	Yes	Frey et al., 2015, Jovel et al., 2016

	Characteristics	16S	WMS	Sources
19	**16S-test. Disadvantage.** The number of 16S copies in genome changes within a wide range (depending on species and even strain).<br>It results in assigned errors when determining taxon representation. Representation of taxons with a larger number of 16S copies in genome will be overestimated, with a smaller number – underestimated.	Yes		Vetrovsky et al., 2013, Tyakht et al., 2014
20	**16S-test. Disadvantage.** The choice of primers for different variable sites (from V1 to V9) to perform amplification, leads to essentially different results not only due to their different characteristics at amplification (affinity), but also due to influence on classification by taxons.	Yes		Jovel et al., 2016, Meisel et al., 2016
21	**16S-test. Disadvantage.** Mutations in variable sites 16S rRNA (from V1 to V9) can interfere with the correct classification by taxons.	Yes		
22	**WMS-test. Disadvantage**. The necessity of maximum elimination of host DNA (hDNA) from biomaterial before sequencing (biochemical methods) and after sequencing (algorithmic methods).		Yes	Ferretti et al., 2017, Frey et al., 2015
	**Cost performance and specific performance data**			
23	Cost at the rate of one sample. Depends on problem statement, number of samples in library, sequencer power and its working mode, protocol of processing sequencing results.	47-60 $	120-290 $	Ranjan et al., 2016, Genohub, Allseq
24	**WMS-test.** In the future, the cost may fall to below $1 per one bacterial genome (2014). It has already happened (see above). The cost can be reduced due to sample preparation or by enrichment of pathogen representation and/or host DNA elimination.		Yes	Applications 2015, Frey et al., 2015
25	Performance time<br>(depends on equipment and problem statement)	2-5 hours	7–60 hours	Frey et al., 2015
26	Requirements to temperature control during transportation and sample preparation.	Lower	Higher	Frey et al., 2015

	Characteristics	16S	WMS	Sources
27	Proven pipeline of test implementation.<br>Term of active use.<br>For 16S-test, however, there are no (and cannot be any) satisfactory schemes for classification to within species (Jovel 2016).	A lot.<br>Over 30 years.	Few.<br>About 10 years.	Sharpton et al., 2014, Ranjan 2016, Nayfach 2016, Vincent et al., 2017, Aransay 2016 (part 12)
28	Number of publications "16S + metagenomic" vs "shotgun + metagenomic"<br>(according to https://scholar.google.com/)	50800	24300	14.02.2019

S2. Resources of metagenomic research and sequencing

Title	Description. Notes
HMP (Human Microbiome Project)	All information about microorganisms, living on and in human body (the project was founded in 2008), contains information on more than 3,000 genomes.
KEGG	Kyoto Encyclopedia of Genes and Genomes (over 4,000 genomes)
MetaHIT	Intestine microbiome. The project was completed in 2012.
Integrated gene catalog (IGC)	Catalog of intestine microbiome genes
NCBI Reference Sequence (RefSeq) Database	NCBI. Reference DB of genomes. Statistics.
NCBI.Genbank	NCBI. DB of genomes ~ 186000 genomes of prokaryotes, including ~ 13500 whole ones.
NCBI Microbial Genomes Resources	NCBI. DB of bacterial genomes. Taxonomical tree.
NCBI. Sequence Read Archive	NCBI. DB of metagenomic projects.
Genomes OnLine Database	DB of genomes.
MG-RAST	DB of metagenomic projects.
Allseq. The Sequencing Marketplace.	Information on sequencers.
Genohub	Information resource on sequencing methods and provider choice. Search of provider.
Science Exchange	Information resource on sequencing methods and provider choice. Search of provider.
Omictools	Search of software for processing biological (including metagenomic) research results
Center for Genomic Epidemiology	Resource for infectious disease specialist
The European Bioinformatics Institute. Metagenomics.	DB of metagenomic projects.

S3. Main researches classification and comparison.

Sampling type	16S-test		WMS-test	
	Psoriatic patients	Healthy persons	Psoriatic patients	Healthy persons
**Serum**	Codoner et al., 2018	Cho et al., 2019; Dong et al., 2019 (low quality)		
**Plasma**	Munz et al., 2010	Païssé et al., 2016 (+); Whittle et al., 2019; Qui et al., 2019;		Dinakaran et al., 2014 (+); Long et al., 2016; Grumaz et al., 2016 (+); Kowarsky et al., 2017 (HP – pregnancy);
**Whole blood, phagocytes**	Okubo et al., 2002 (monocytes – without metagenome, +);	Païssé et al., 2016 (and its fractions, +); Lelouvier et al., 2016 (leukocytic mass, +); Gosiewski et al., 2017; Panaiotov et al., 2018; Puri et al., 2018 (+); Li et al., 2018 (including neutrophils, +); Qian et al., 2018 (leukocytic mass, +), Shah et al., 2019 (leukocytic mass, +); Serena et al., 2019;	NCS1 (in future, +)	NCS1 (in future, +)

Note: + - concentration was (will be) determined.

Main researches in which metagenome is defined and also several key works on psoriasis can be grouped in two key parameters: biomaterial sampling type (serum or plasma or whole blood, phagocytes) and sequencing technique (16S-test or WMS-test) (See table above).

Blood serum metagenome of psoriatic patients was defined once and as it is possible to judge by results, unsuccessfully (Ramírez-Boscá et al., 2015, Codoner et al., 2018). Metagenome has not been found neither in one of 27 HP, nor in 75% from more than 50 PP. Similar problems have arisen in work (Dong et al., 2019), metagenome has been defined only at 101 of 311 inspected. On the other hand in work (Cho et al., 2019) metagenome has been defined at all inspected (324 patients and 402 HP).

Can be one of reasons of failures the wrong preparation and/or storage of biomaterial before DNA isolation. All or most part of bacDNA will be lost if not to provide maximum termination of its degradation after blood sampling. The most widespread technology of receiving serum presumes staying of blood for 15-30 minutes at room temperature for coagulation and subsequent centrifuging at low temperature. If this protocol for some samples lasts longer and/or is carried out without necessary cooling, then enzymes will degrade bacDNA and blood phagocytes will endocyte it. In work (Cho et al., 2019) requirements of this protocol have been completely fulfilled for all samples.

In the majority of researches metagenome was defined for plasma (Dinakaran et al., 2014, Long et al., 2016, Grumaz et al., 2016, Kowarsky et al., 2017, Whittle et al., 2019). After publication of pioneer work (Païssé et al., 2016), results of several more researches in which whole blood metagenome was also defined have been published (Lelouvier et al., 2016, Gosiewski et al.,

2017, Panaiotov et al., 2018, Puri et al., 2018, Li et al., 2018, Qian et al., 2018, Shah et al., 2019, Serena et al., 2019).

In some of these works concentration of bacDNA in whole blood (noted +) was defined that has allowed to compare for the first time these data for healthy persons (Table 2 in main text). In all these works metagenome was defined by 16S-test and, therefore, only to within genus of bacteria.

Only in these works the possibility of comparison of absolute bacDNA concentration (as in general, and for separate genera) has appeared at disease with control healthy group.

WMS-test allows to define metagenome to within species of bacteria. Unfortunately it has been made only for plasma (Dinakaran et al., 2014, Long et al., 2016, Grumaz et al., 2016, Kowarsky et al., 2017). In work (Dinakaran et al., 2014) WMS-test has been executed only for 3 patients and 3 HP that it is obviously not enough for statistically significant conclusions. In work (Long et al., 2016) bacDNA concentration was not defined and contamination level was not estimated that does its results less significant.

In work (Kowarsky et al., 2017) effective and versatile control of contamination level has been applied, but bacDNA concentration was not defined. Results of (Grumaz et al., 2016) in which concentration of all cell-free DNA was defined that indirectly allows to estimate plasma bacDNA concentration are most interesting.

Before WMS-test performance for obtaining information about metagenome it is reasonable to carry out preliminary elimination of host DNA (Opota et al., 2015, Yigit et al., 2016, Marotz et al., 2018). It allows to increase significantly % of reads of non-host DNA (including bacDNA) in results of sequencing and by that to increase their reliability. Most effectively such elimination is carried out by NebNext Microbiome DNA Enrichment. However in works using WMS-test the preliminary elimination of host DNA was not carried out and, as a result, from 96% to 99,5% of reads were mapped on human genome.

In future researches with sequencing it is reasonable to obtain information about metagenome to within species, i.e. by WMS-test. BacDNA concentration in whole blood is three orders higher in comparison with its concentration in plasma. Therefore as biomaterial it is necessary to use whole blood (optimum - postprandial). It will provide definition of values of bacDNA concentration for minority species above potential contamination level (Païssé et al., 2016). Assessment of contamination level has to be most exact and correct (Kowarsky et al., 2017). Before WMS-test performance for definition of whole blood metagenome it is necessary to carry out the greatest possible elimination of host DNA. As a result of such research significant and detailed information about blood metagenome at any diseases will be obtained.

Metagenome of plasma or whole blood of psoriatic patients in one of works was not defined. WMS-test was not used for studying of any fraction of blood of psoriatic patients. About bacDNA concentration in blood monocytes of psoriatic patients it is possible to judge only by work (Okubo et al., 2002). In future researches of psoriatic patients it is necessary to define whole blood metagenome by WMS-test, and also bacDNA concentration. Only such approach will give the chance to reveal correlation of psoriasis severity with total bacDNA concentration of presumed psoriagenic and/or pathogenic species (Supplement S5).

S4. Income of bacterial products into blood flow from small intestine.

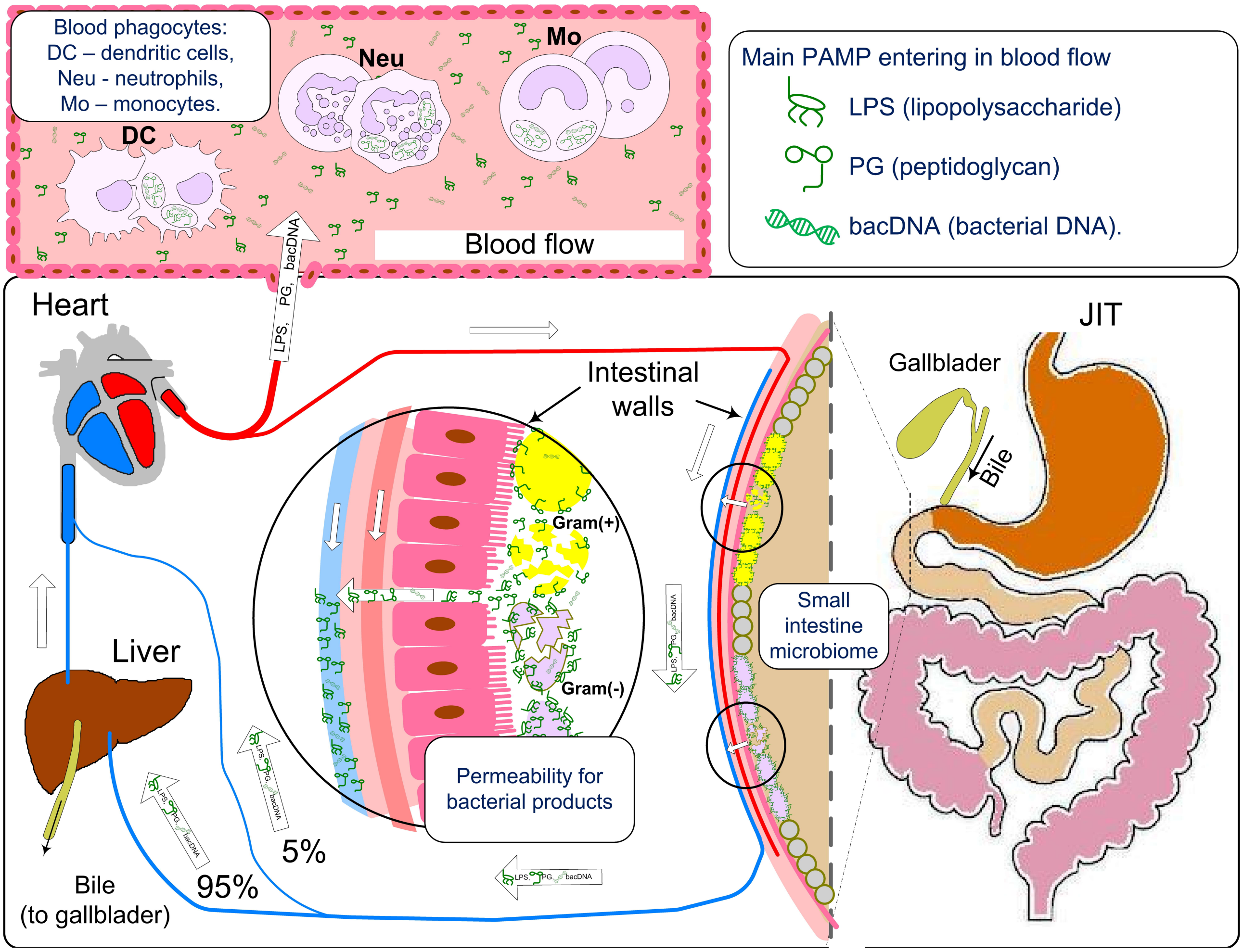

Fig. S1. Income of bacterial products into blood flow from small intestine.

Intestine microbiome self-renews; as a result of bacterial activity and death of bacterial cells, bacterial products develop, particularly LPS (lipopolysaccharide), cellular wall component of Gram(-) bacteria and PG (peptidoglycan), the main cellular wall component of Gram(+) bacteria, as well as bacDNA (bacterial DNA). At normal, and especially at increased small intestine permeability for bacterial products, their essential part gets into systemic blood flow.

At least 95% of venous blood from small intestine passes by portal vein through liver, where an essential part of bacterial products is degraded and filtered (and subsequently returns into small intestine together with bile). Due to porto-caval anastomosis, though, up to 5% of venous blood bypasses liver and gets directly into the system of superior vena cava, and then into systemic blood flow (including non-degraded bacterial products).

In systemic blood flow, bacterial products are constantly utilized, mainly with phagocyte participation (primarily neutrophils). The figure shows both young phagocytes (which have just left bone marrow and therefore do not contain bacterial products) and those which have already endocyted bacterial products. At each timepoint most non-degraded bacterial products in systemic blood flow are either bound by phagocytes through surface receptors or are endocytosed and located in phagocytes.

S5. YN-model of psoriasis pathogenesis. Partial description.

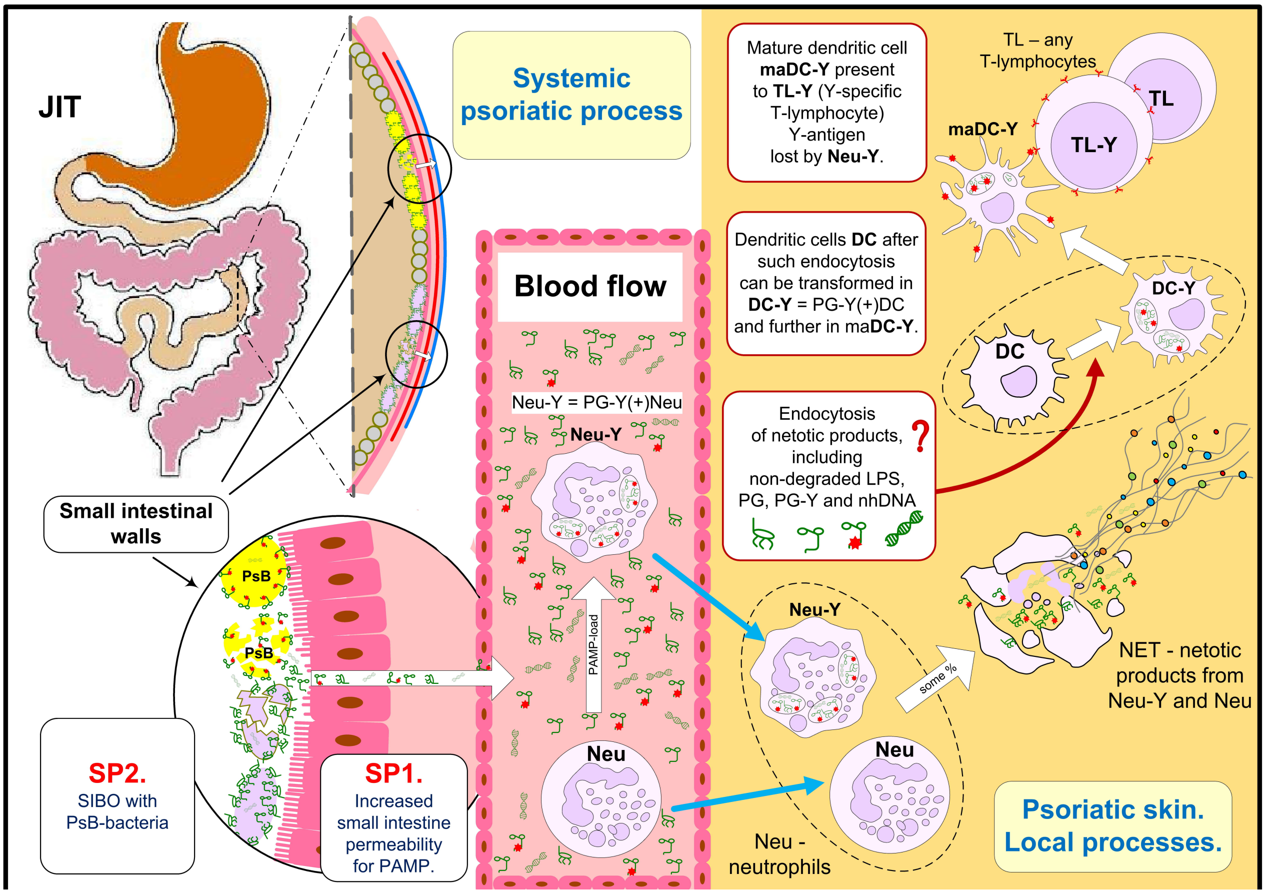

Fig. S2. YN-model of psoriasis pathogenesis. (Peslyak and Korotky 2019, part 5.2).

**IB-Y**	Interpeptide bridges of peptidoglycan *Str.pyogenes*: (L-Ala)-(L-Ala) or (L-Ser)-(L-Ala).
**PG-Y**	Peptidoglycan A3alpha with interpeptide bridges IB-Y (but can also contain others bridges)
**PsB**	Psoriagenic bacteria - species of bacteria presumed psoriagenic (with PG-Y peptidoglycan)
**Y-antigen**	part(s) of interpeptide bridge IB-Y
**SIBO**	Small intestine bacterial overgrowth. Excess of total bacteria concentration over norm and/or pathogens presence in biomaterial. Smears, scrapes from mucosa or aspirates can be used as biomaterial.

**Systemic psoriatic process**	**SP1.** Increased small intestine permeability for PAMP (including LPS, PG, bacDNA).<br>**SP2.** SIBO with PsB-bacteria.<br>SP1 and SP2 result in chronically increased<br>- concentrations of PAMP (including PG-Y) in blood flow;<br>- PAMP- and (PG-Y)-load on blood neutrophils;<br><br>As a result, many blood neutrophils<br>- become PAMP- and (PG-Y)-carriers;<br>- pass into prenetotic state;<br>- undergo netosis.

**Local processes**	In healthy skin neutrophils are practically absent. They are attracted from blood flow at the earliest stage of psoriatic plaque initiation (even before visible skin changes). Their intensive attraction continues as long as the plaque is present. In stable or growing plaque, neutrophils end their existence due to pro-inflammatory environment, mostly by netosis (or, at plaque remission, by apoptosis).<br><br>Non-degraded PAMP (including PG-Y) brought from blood flow get into netotic products. They are endocyted by skin phagocytes, particularly by dendritic cells.<br>Dendritic cells process PG-Y and present Y-antigen (contained in PG-Y) to effector T-lymphocytes. Other PAMP act as adjuvants. False adaptive response of skin immune system to false PsB-infection is formed. Psoriatic plaques appear and grow while **systemic psoriatic process** is going on, i.e. while neutrophils still attracted from blood flow are abundant in PAMP and PG-Y.

References for supplements

Applications of Clinical Microbial Next-Generation Sequencing. (2016). Report on an American Academy of Microbiology Colloquium held in Washington, DC, in April 2015. p.56. link.

Aransay AM, Trueba JLL. (2016). Field Guidelines for Genetic Experimental Designs in High-Throughput Sequencing. Springer International Publishing Switzerland, 399 p. ISBN: 978-3-319-31350-4.

Bhat S, Emslie KR. (2016). Digital polymerase chain reaction for characterisation of DNA reference materials. Biomol Detect Quantif. 10:47-49. doi:10.1016/j.bdq.2016.04.001

Cho EJ, Leem S, Kim SA, Yang J, Lee YB, Kim SS, et al. (2019). Circulating Microbiota-Based Metagenomic Signature for Detection of Hepatocellular Carcinoma. Sci Rep. 9:7536. doi:10.1038/s41598-019-44012-w

Codoñer FM, Ramírez-Bosca A, Climent E, Carrión-Gutierrez M, Guerrero M, Pérez-Orquín JM, et al. (2018). Gut microbial composition in patients with psoriasis. Sci Rep. 8:3812. doi:10.1038/s41598-018-22125-y

Dinakaran V, Rathinavel A, Pushpanathan M, Sivakumar R, Gunasekaran P, Rajendhran J. (2014). Elevated levels of circulating DNA in cardiovascular disease patients: metagenomic profiling of microbiome in the circulation. PLoS One. 9:e105221. doi:10.1371/journal.pone.0105221

Dong Z., Chen B.,Wang D. et al. (2018). Serum Microbiome Analysis as a Tool Towards Targeted Biomarker for Gastric Cancer [Preprint]. Available at: https://ssrn.com/abstract=3284853 (Accessed Nov 16, 2018)

Ferretti P, Farina S, Cristofolini M, Girolomoni G, Tett A, Segata N. (2017). Experimental metagenomics and ribosomal profiling of the human skin microbiome. Exp Dermatol. 26:211-219. doi:10.1111/exd.13210

Frey KG, Bishop-Lilly KA. (2015). Next-Generation Sequencing for Pathogen Detection and Identification. Chapter 15 in Methods in Microbiology. 42:525–554. ISSN 0580-9517. link.

Glassing A, Dowd SE, Galandiuk S, Davis B, Chiodini RJ. (2016). Inherent bacterial DNA contamination of extraction and sequencing reagents may affect interpretation of microbiota in low bacterial biomass samples. Gut Pathog. 8:24. doi:10.1186/s13099-016-0103-7

Gosiewski T, Ludwig-Galezowska AH, Huminska K, Sroka-Oleksiak A, Radkowski P, Salamon D, et al. (2017). Comprehensive detection and identification of bacterial DNA in the blood of patients with sepsis and healthy volunteers using next-generation sequencing method - the observation of DNAemia. Eur J Clin Microbiol Infect Dis. 36:329-336. doi:10.1007/s10096-016-2805-7.

Grumaz S, Stevens P, Grumaz C, Decker SO, Weigand MA, Hofer S, et al. (2016). Next-generation sequencing diagnostics of bacteremia in septic patients. Genome Med. 8:73. doi:10.1186/s13073-016-0326-8

Jovel J, Patterson J, Wang W, Hotte N, O'Keefe S, Mitchel T, et al. (2016). Characterization of the Gut Microbiome Using 16S or Shotgun Metagenomics. Front Microbiol. 7:459. doi:10.3389/fmicb.2016.00459

Kowarsky M, Camunas-Soler J, Kertesz M, De Vlaminck I, Koh W, Pan W, et al. (2017). Numerous uncharacterized and highly divergent microbes which colonize humans are revealed by circulating cell-free DNA. Proc Natl Acad Sci U S A. 114:9623-9628. doi:10.1073/pnas.1707009114

Lelouvier B, Servant F, Païssé S, Brunet AC, Benyahya S, Serino M, et al. (2016). Changes in blood microbiota profiles associated with liver fibrosis in obese patients: A pilot analysis. Hepatology.64:2015-2027. doi:10.1002/hep.28829

Li Q, Wang C, Tang C, Zhao X, He Q, Li J. (2018). Identification and Characterization of Blood and Neutrophil-Associated Microbiomes in Patients with Severe Acute Pancreatitis Using Next-Generation Sequencing. Front Cell Infect Microbiol. 8:5. doi:10.3389/fcimb.2018.00005

Long Y, Zhang Y, Gong Y, Sun R, Su L, Lin X, et al. (2016). Diagnosis of Sepsis with Cell-free DNA by Next-Generation Sequencing Technology in ICU Patients. Arch Med Res. 47:365-371. doi: 10.1016/j.arcmed.2016.08.004.

Marotz CA, Sanders JG, Zuniga C, Zaramela LS, Knight R, Zengler K. (2018). Improving saliva shotgun metagenomics by chemical host DNA depletion. Microbiome. 27;6(1):42. doi:10.1186/s40168-018-0426-3

Meisel JS, Hannigan GD, Tyldsley AS, SanMiguel AJ, Hodkinson BP, Zheng Q, Grice EA. (2016). Skin Microbiome Surveys Are Strongly Influenced by Experimental Design. J Invest Dermatol. 136:947-56. doi:10.1016/j.jid.2016.01.016

Munz OH, Sela S, Baker BS, Griffiths CE, Powles AV, Fry L. (2010). Evidence for the presence of bacteria in the blood of psoriasis patients. Arch Dermatol Res. 302:495-8. doi:10.1007/s00403-010-1065-0

Nakatsuji T, Chiang HI, Jiang SB (2013). The microbiome extends to subepidermal compartments of normal skin. Nat Commun. 4:1431. doi:10.1038/ncomms2441

Nayfach S, Pollard KS. (2016). Toward Accurate and Quantitative Comparative Metagenomics. Cell. 166:1103-1116. doi:10.1016/j.cell.2016.08.007

Okubo Y, Oki N, Takeda H, Amaya M, Ito S, Osada M, et al. (2002). Increased microorganisms DNA levels in peripheral blood monocytes from psoriatic patients using PCR with universal ribosomal RNA primers. J Dermatol. 29:547-55. doi:10.1111/j.1346-8138.2002.tb00179.x.

Opota O, Jaton K, Greub G. (2015). Microbial diagnosis of bloodstream infection: towards molecular diagnosis directly from blood. Clin Microbiol Infect. 21:323-31. doi:10.1016/j.cmi.2015.02.005

Païssé S, Valle C, Servant F, Courtney M, Burcelin R, Amar J, Lelouvier B. (2016). Comprehensive description of blood microbiome from healthy donors assessed by 16S targeted metagenomic sequencing. Transfusion. 56:1138-47. doi:10.1111/trf.13477

Ranjan R, Rani A, Metwally A, McGee HS, Perkins DL. (2016). Analysis of the microbiome: Advantages of whole genome shotgun versus 16S amplicon sequencing. Biochem Biophys Res Commun. 469:967-77. doi:10.1016/j.bbrc.2015.12.083

Panaiotov S, Filevski G, Equestre M, Nikolova E, Kalfin R (2018). Cultural Isolation and Characteristics of the Blood Microbiome of Healthy Individuals. Advances in Microbiology, 8:406-421. doi:10.4236/aim.2018.85027

Peslyak MY, Korotky NG. (2019). Metagenomes of blood and psoriatic skin. Research project., Moscow, Antipsoriatic Association "The Natural Alternative", ISBN 9785905504068, doi: 10.5281/zenodo.2667679

Puri P, Liangpunsakul S, Christensen JE, Shah VH, Kamath PS, Gores GJ, et al. (2018). The circulating microbiome signature and inferred functional metagenomics in alcoholic hepatitis. Hepatology. 67:1284-1302. doi:10.1002/hep.29623

Ramírez-Boscá A, Navarro-López V, Martínez-Andrés A, Such J, Francés R, Horga de la Parte J, Asín-Llorca M. (2015). Identification of Bacterial DNA in the Peripheral Blood of Patients With Active Psoriasis. JAMA Dermatol. 151:670-1. doi:10.1001/jamadermatol.2014.5585.

Qian Y, Yang X, Xu S, Wu C, Qin N, Chen SD, Xiao Q. (2018). Detection of Microbial 16S rRNA Gene in the Blood of Patients With Parkinson's Disease. Front Aging Neurosci. 10:156. doi:10.3389/fnagi.2018.00156

Qiu J, Zhou H, Jing Y, Dong C. (2019). Association between blood microbiome and type 2 diabetes mellitus: A nested case-control study. J Clin Lab Anal. 33:e22842. doi:10.1002/jcla.22842

Serena G, Davies C, Cetinbas M, Sadreyev RI, Fasano A. (2019). Analysis of Blood and Fecal Microbiome Profile in Patients with Celiac Disease. Human Microbiome Journal. 11:100049 doi:10.1016/j.humic.2018.12.001

Shah NB, Allegretti AS, Nigwekar SU, Kalim S, Zhao S, Lelouvier B, et al. (2019). Blood Microbiome Profile in CKD : A Pilot Study. Clin J Am Soc Nephrol. 14:692-701. doi:10.2215/CJN.12161018

Sharpton TJ. (2014). An introduction to the analysis of shotgun metagenomic data. Front Plant Sci. 5:209. doi:10.3389/fpls.2014.00209

Stevenson M, Pandor A, Martyn-St James M, Rafia R, Uttley L, Stevens J, et al. (2016). Sepsis: the LightCycler SeptiFast Test MGRADE®, SepsiTest™ and IRIDICA BAC BSI assay for rapidly identifying bloodstream bacteria and fungi - a systematic review and economic evaluation. Health Technol Assess. 20:1-246. doi:10.3310/hta20460

Tan B, Ng C, Nshimyimana JP, Loh LL, Gin KY, Thompson JR. (2015). Next-generation sequencing (NGS) for assessment of microbial water quality: current progress, challenges, and future opportunities. Front Microbiol. 6:1027. doi:10.3389/fmicb.2015.01027

Tyaht AV. (2013). Functional analysis of human intestinal metagenome. Dissertation. Moscow, 131 p., (rus), link

Vetrovsky T, Baldrian P. (2013). The variability of the 16S rRNA gene in bacterial genomes and its consequences for bacterial community analyses. PLoS One. 8:e57923. doi:10.1371/journal.pone.0057923

Vincent AT, Derome N, Boyle B, Culley AI, Charette SJ. (2017). Next-generation sequencing (NGS) in the microbiological world: How to make the most of your money. J Microbiol Methods. 138:60-71. doi:10.1016/j.mimet.2016.02.016

Whittle E, Leonard MO, Harrison R, Gant TW, Tonge DP. (2019). Multi-Method Characterization of the Human Circulating Microbiome. Front Microbiol. 9:3266. doi:10.3389/fmicb.2018.03266

Yigit E, Feehery GR, Langhorst BW, Stewart FJ, Dimalanta ET, Pradhan S, et al. (2016). A Microbiome DNA Enrichment Method for Next-Generation Sequencing Sample Preparation. Curr Protoc Mol Biol. 115:7.26.1-7.26.14. doi:10.1002/cpmb.12
